# Case of Encephalitis, with Ossific Deposites in the Membranes of the Brain

**Published:** 1849-09

**Authors:** Thomas Hall

**Affiliations:** Toulon, Illinois


### ARTICLE III.

*Case of Encephalitis, with Ossific Deposites in the Membranes of the Brain.* By THOMAS HALL, M. D., M. R. C. S., of Toulon, Illinois.

William B. Maccanse, the subject of the disease, was 25 years old, of sanguine and nervous temperament, and strumous diathesis. His parents as far as can be ascertained, were healthy, and his brother and sister who are living here, appear to have good average constitutions, and enjoy generally good health.

About four months ago the patient lost his wife, and the next time his brother saw him, (a few weeks afterward,) he noticed an altered appearance of the eyes. With the exception of the ague his previous health was good. He had been subject from his seventh year to bleeding from the nose, which had come on more frequently during the past year, than the two preceeding years. His complxction for several months had been more florid than usual, and had perceptibly increased during the past winter. He had often complained of head-ache for three months past, which was aggravated whenever he took cold. He was naturally of a merry jovial turn, but latterly taciturn and desponding—often remarked, if reading any length of time, that the letters danced before his eyes—the powers of comparison, memory, attention—naturally great, were debilitated, and three days before he was prostrated, he failed in telling an oft told tale, and made remarks about the failure himself—was fond of telling anecdotes and relating stories but had nearly quit it, walked slower than usual and was inclined to sit still and be quiet.

April 5th. He complained of severe head-ache and fever, and applied for medical advice. He was given a dose of calomel and rhubarb, which operated severely, and partially relieved him.

April 6th. Felt heaviness at the stomach, for which took some cough-drops at his own suggestion; a light vomiting was produced, and the only time it occurred during the continuance of the disease.

7th. Took, on his own judgement, a sweat of red pepper tea.

8th. Took another dose of calomel and rhubarb, followed by castor-oil, which operated so severely that his medical adviser, who saw him during its action, thought it best to check it with opium. Eight doses of calomel were left, one to be taken every three hours, with a dose of oil between. The discharges from the bowels were dark and billious. The prominent symptoms—head-ache and fever.

9th. His friends thought him somewhat flighty, he told them that his head was dizzy, although he expressed himself as feeling better when sitting up than lying down—freely perspiring—countenance florid—the unnatural appearance of the eyes increasing, with an expression wild and vacant, and he answers questions with hesitation. The fæces in color natural. Calomel and ipecac to to be taken every four hours.

10th. Now answers hesitatingly and irrelevantly; five doses of calomel and Dover's Powders, one to be taken every three hours up to this date. Head-ache and fever were principally complained of, gradually increasing, with occasional remissions. The disease was pronounced a mild form of billious fever.

11th. This morning apparently better, sat up half an hour, walked into an adjoining room, undressed and went to bed; he soon however sent for his brother to dress him. About ten o'clock began to feel weak, seemed anxious to speak but could not; he shortly rallied and told his brother he was sinking, for which he took two table spoonsful of pepper-sauce which was repeated in twenty minutes. His brother now left him but in from five to ten minutes was sent for, when he appeared to be in some kind of a fit. The neighbors present thought it a congestive chill, for which they vigorously rubbed him with salt and water, and poured cold water on his head from a tea-pot. At the commencement of this paroxysm, his medical adviser was sent for, but before he arrived reaction had taken place and the patient was freely perspiring. Morphine and Piperine were now given in red-pepper tea every two or three hours until seven doses were taken. He was evidently deranged, difficulty of swallowing commencing, particularly in regard to fluids. The Prognosis was favorable, and a dose of oil ordered to move the bowels.

12th. Derangement continues, strength declining, and still sweating. Dovers powders and calomel were given every three hours. The bowels moved two or three times and he appeared to be conscious of their action. About three o'clock P. M., he could not swallow, but at ten a dose of medicine was given with some difficulty. A favorable prognosis is confidently given, "the disease is now headed," "the difficulty of swallowing is nothing but obstinacy." The opinion of the friends in regard to the issue is decidedly unfavorable.

13th. Attempts to give medicine not succeeding, the friends suggested external applications. "There is no way of combating the disease but by calomel," and nothing was done for several hours. The disease is pronounced a "billious form of fever, and a very simple case at that—the head partaking of the disorder." "I have fired bomb-shells at the stomach, without any benefit, and something else must be tried." During the after part of the day the throat, nape of the neck, sternum and abdomen were blistered, sinapisms were applied to the upper and lower extremeties, (probably on the supposition that by firing a profusion of shot, one might possibly hit.) A fatal prognosis is now given, and an acknowledgement of being beaten, but "if three more doses of medicine could be introduced into the stomach, the patient could be saved.

14th. Early this morning the patient is given up. The history thus far has been derived from the patients brother and brother-in-law. The verbiage being retained as nearly as possible. and every one must admire its clearness; of its accuracy there can be no doubt.

Dr. Chamberlain and myself were sent for and saw him along with Dr. Smith, (who had not previously seen the case,) at three o'clock P. M. The patient was restless, rolling from side to side—the head hot—eye-brows knit—eyes suffused—pupils dilated, but not equally, the left more than the right; they responded but in a trifling degree to light, but the right more than the left—the countenance expressive of alarm with a wild and vacant gaze—the carotids beat powerfully and firmly—the radials barely perceptible—pulse 108, inability to speak and swallow—the tongue dry, brown in the centre and red at the tips and edges, and the extremities cold.

Diagnosis, inflammation of the brain and membranes. Prognosis, based more on the duration of the disease than the intensity of the inflammation, was unfavorable.

Treatment. Cupping to the extent of eight ounces from the back of the neck, blister, to the spine, cold to the head and sinapism to the lower extremities. There was no manifestation of feeling when the scarificator was applied, but he flinched on the application of the fourth and fifth glasses. An injection of gruel and croton oil was administered, and after the cupping he took a dose of calomel and some water without much difficulty.

Eight o'clock P. M., appeared better. Cupped again and took a dose of calomel. The injection was repeated.

15th.—6 o'clock A. M. Appearances more favorable. Head cooler, less knitting of the brow, tongue cleaner, moister and protruded without much difficulty, the radial artery fuller, the carotids weaker, pulse 112, the lower extremities warmer, more tranquil, had taken fluids frequently, and the bowels had acted twice during the night: an injection of oil and turpentine was administered. Nine o'clock, cupped again, treatment continued. Eight o'clock P. M., pulse 80 and weak, patient sinking. Although the case was hopeless, constnt but ineffectual efforts were used during the night to rouse the vital energies—he died at sunrise, the next moning.

Post-mortem 9 hours after death. Body cold, not much emaciated. The chest and abdomen first opened. The organs in the chest perfectly healthy. The spleen was about four times in normal size and somewhat indurated—the mesenteric glands were large and redder than natural, but no scrofulous deposits in them—there was a small invagination of the ileum, the other organs in this cavity were sound. The head was opened carefully with a saw. The dura-matter separated easily, and was free from disease as well as its arachnoid lining, except the falciform process, which was inflamed, and had on its surface several ossific deposits, one of a considerable size had made a sulcus on the left lobe of the cerebrum, and numerous deposits of the same character could be distinctly felt, like grains of sand, on the same process. These deposits of any size, were seven in number, one about half an inch long, by a quarter wide, and a little over an 8th in thickness, hard in texture, rough and pointed on the surface, and were covered by the arachnoid, except the lragest which had caused absorption of the dura-mater and its lining membrane and was pressing against the arachnoid covering of the pia-mater. The sinuses were gorged with blood. The arachnoid of the pia-mater opake in places, with opalescent fluid underneath other portions which had preserved their transparency, and was partially adherent to the pia-mater, which was inflamed; its arteries could be distinctly traced and its veins were distended. There were five adhesions of considerable size of this membrane to the brain, requiring the knife to separate them; and the convolutions, at least many of them, were in the same condition. The brain had evident marks of inflammation, and was highly congested; there was but a small amount of fluid in the ventricles, the cerebellum and lower surface of the brain healthy. The left cervical glands were enlarged, no other abnormal appearance detected.

The history of the case, sufficiently full for practical purposes, is replete with interest. There is little doubt but that twelve months ago, disordered or diseased action commenced in the brain or its membranes; and that grief and emotion of some kind was the exciting cause. Altered expression of the eyes—debilitated intellect—the countenance becoming more florid, notwithstanding the nose bled more frequently and profusely—intermittent head-ache often severe, and for the last two months nearly constant—and the change in manner from great cheerful nees to gloom and despondency, were sufficient to create anxiety in any Physician at all conversant with cerebral disease, and to guide him in forming an opinion and regulating the treatment when the disease was fully formed and developed in all its fearful energies. And in this case there was no disordered function of distant organs to divert the attention of a physician from the seat of the disease, or to account, by sympathy, for the disturbance in the head.

There can be no doubt that the ossific deposites would cause occasional, if not constant, disturbances; they would act as so many centres of irritation that a common additional exciting cause would increase the irritation,—congestion greater or less in degree would follow—and hence the intermittent head-ache. It is probable that during the night of the 8th, if not previously, inflammation of the membranes commenced, limited, may be in extent to parts contiguous to the deposits, and that it gradually increased in size and intensity until the morning of the 11th, when the brain became involved in the morbid action.

The diagnosis was easy. That inflammation existed within the head admitted not of a doubt,---the extent of it, however, was not so palpable. We were all convinced that the membranes were inflamed, and that it extended to the brain was inferred, principally from the inability to swallow. [Attributed by our predecessor to obstinacy.] Difficult deglutition and the total loss of the power of swallowing, were looked upon as severe indications of cerebral inflammation, when there was no local lesions to account for them. During my studentship, I had frequent opportunities of seeing the diagnosis verified; but in examining the subject since this patient's death, I find no allusion to the special bearing of the symptoms, except in Dr. Copland's Medical Dictionary [London Edition], Article, Brain, diseases of, Page 233 § 180. "In both these states of the disease, the difficulty of swallowing is great, so that fluids are sometimes regurgitated by the nose; and when the substance of the brain is chiefly affected, deglutition is often nearly, or altogether, abolished in the most intense cases." I have no doubt of the correctness of the above extract---my own observations extending back some 30 years have proved its truth and its value as a diagnostic sign, and worthy of the few remarks here made.

When the diagnosis is erroneous, the treatment cannot, except by accident, be good. The merest tyro in medicine will at once see that the administration of opium and its preparations in a case like the above, must be followed by disastrous consequences. The calomel was given with the intention of salivating the patient; a practice too frequently adopted, and when accomplished, looked upon by some physicians as a proof of superior science—the ne plus ultra of professional skill. It may be so, but salivation is no God-send for a man with a scrofulous constitution. The purging was good and so was the application of the cold water by the friends on the morning of the 11th. We used calomel, not as a mercurial, but as a purgative, the most convenient, and as being tasteless and in a small compass, the most likely to be taken without irritating the patient. The moral treatment is frequently more important than the medicinal, and in every case should be associated. Gentleness, kindness ought, at all times to be manifested by a physician to the sick, and especially when the disease is irritation or inflammation of the brain and its membranes. But it is not all that think so. Some believe, or act as if they believed, that cursing at the bedside, like salivation, is another proof of superior knowledge. I have trespassed already too much on your columns, but allow me a short space to make a few remarks or post-mortem investigations---I never urge it except in unusual cases---and have never been refused. In this instance the friends were more than satisfied: it was a great consolation to them under the circnmstances---to know that under the most judicious treatment, as the cause could not with certainty be divined and never removed, death must have been the inevitable result---if not during this attack, at no distant date. In our examinations we are careful to leave the body as little disfigured as possible, and in this case we overcame one of the greatest causes by drilling four holes through the parietal bones, one on each side of the saw-mark, passing small wire through them, and when the skull cap was accurately adjusted, twisting them tight---the cap was held firmly in its place, and when the integuments were returned, the forehead was as smooth and level as before. We object to no one being present---and some evidently get interested in the investigation and speak warmly in favour of it. By pursuing this open, honorable course, and by treating the dead with all respect---the feeling of the community is decidedly right on this necessary and instructive proceeding, which tells something for the Physicians, but more for the intelligence of our county. When our diagnosis is proved incorrect, though humiliating, it is useful by stimulating us to closer observation and more rigid scrutiny in obscure cases---and when correct, we feel the truth of the sentiment so beautifully expressed by the Roman poet.

"Felix qui potuit rerum cognoscere causas."

